# A Comprehensive Analysis of Fel Ursi and Its Common Adulterants Based on UHPLC-QTOF-MS^E^ and Chemometrics

**DOI:** 10.3390/molecules29133144

**Published:** 2024-07-02

**Authors:** Xianrui Wang, Haonan Wu, Minghua Li, Xiaohan Guo, Xianlong Cheng, Wenguang Jing, Feng Wei

**Affiliations:** 1 Institute for Control of Chinese Traditional Medicine and Ethnic Medicine, National Institutes for Food and Drug Control, Beijing 102629, China; niuyun006097@163.com (X.W.); 19861403424@163.com (H.W.); liminghua@nifdc.org.cn (M.L.); guoxiaohan@nifdc.org.cn (X.G.); weifeng@nifdc.org.cn (F.W.); 2 Faculty of Functional Food and Wine, Shenyang Pharmaceutical University, Shenyang 110016, China

**Keywords:** chemical marker, Fel Ursi, UHPLC-QTOF-MS^E^, multivariate statistical analysis, PCA, OPLS-DA, difference analysis, Xiong Dan

## Abstract

Background: As one of the four most valuable animal medicines, Fel Ursi, named Xiong Dan (XD) in China, has the effect of clearing heat, calming the liver, and brightening the eyes. However, due to the special source of XD and its high price, other animals’ bile is often sold as XD or mixed with XD on the market, seriously affecting its clinical efficacy and consumers’ rights and interests. In order to realize identification and adulteration analysis of XD, UHPLC-QTOF-MS^E^ and multivariate statistical analysis were used to explore the differences in XD and six other animals’ bile. Methods: XD, pig gall (Zhu Dan, ZD), cow gall (Niu Dan, ND), rabbit gallbladder (Tu Dan, TD), duck gall (Yan Dan, YD), sheep gall (Yang Dan, YND), and chicken gall (Ji Dan, JD) were analyzed by UHPLC-QTOF-MS^E^, and the MS data, combined with multivariate analysis methods, were used to distinguish between them. Meanwhile, the potential chemical composition markers that contribute to their differences were further explored. Results: The results showed that XD and six other animals’ bile can be distinguished from each other obviously, with 27 ions with VIP > 1.0. We preliminarily identified 10 different bile acid-like components in XD and the other animals’ bile with significant differences (*p* < 0.01) and VIP > 1.0, such as tauroursodeoxycholic acid, Glycohyodeoxycholic acid, and Glycodeoxycholic acid. Conclusions: The developed method was efficient and rapid in accurately distinguishing between XD and six other animals’ bile. Based on the obtained chemical composition markers, it is beneficial to strengthen quality control for bile medicines.

## 1. Introduction

Fel Ursi, named Xiong Dan (XD) in China, is the dried product obtained by draining the bile of Selenarctos thibetanus G Cuvier, *Ursus arctos* L. through gallbladder surgery [1,2]. XD has efficacy in clearing away heat and detoxifying and is anti-bacterial, anti-inflammatory, and liver-protective [3]. It is commonly used in traditional Chinese medicine and is also an important raw material for producing a variety of proprietary Chinese medicines. XD mainly contains bile acids, amino acids, bile pigments and fat, phospholipids, and trace elements. Recent research has shown that as a potential new anticancer drug, XD can exert anticancer properties in FRO cells by regulating the Akt/mTOR signaling pathway to induce apoptosis and inhibit angiogenesis [4]. Although the medicinal value of XD is rich, due to its scarcity and high price, the XD on the market often appears as counterfeit or adulterated products [5]. The common forgeries are cow gallbladder (Niu Dan, ND), sheep gallbladder (Yang Dan, YD), pig gallbladder (Zhu Dan, ZD), and other animals’ gallbladder (such as chicken gallbladder (Ji Dan, JD), rabbit gallbladder (Tu Dan, TD), duck gallbladder (Ya Dan, YD), etc.) [6]. Among them, ND, ZD, YD, and JD are commonly used as adulterants because they are easier to obtain and have similar properties to bear bile. This kind of adulteration phenomenon leads to the uneven quality of XD on the market, which seriously affects its clinical efficacy and consumer rights and interests; therefore, the authenticity and detection of the adulteration of XD is of great significance for the quality control of XD.

In order to identify XD and its counterfeits, related researchers have carried out a large number of studies and established a variety of identification methods. For example, traditional empirical identification mainly includes the visual method, the hand-rubbing method, the mouth-tasting method, the water testing method, the fire method, etc. [6]. Moreover, thin-layer chromatography, fluorescence analysis, microscopic identification, electrophoretic identification, and X-ray diffraction have all been applied to the identification of XD [6,7]. Xiong J et al. established the fingerprints of bile medicines using HPLC and ELSD [8]. Based on an electronic tongue (E-tongue), an electronic nose (E-nose), and GC-MS, Kelu Lei et al. detected tauroursodeoxycholic acid (TUDCA) and taurochenodeoxycholic acid (TCDCA) in XD and constructed an identification model based on the Random Forest algorithm [9]. In addition, Qiao X et al. used liquid chromatography coupled with triple quadrupole mass spectrometry to determine the content of several bile acids in XD, ND, and ZD and applied this to quality control [10].

All the above studies have helped to somewhat strengthen the quality control and market supervision of bear bile. However, there are still some limitations: (1) for traditional identification, methods such as hand-rubbing, water testing, microscopic identification, etc., need professionally experienced personnel for identification, and their subjectivity makes it difficult for them to become a legal standard; (2) compared with mass spectrometry, the precision of E−noses, E−tongues, and high-performance liquid chromatography (HPLC) is relatively low, and most of the studies have been limited to the analysis of counterfeit ND, ZD, and YND and did not include a variety of bile components in a unified analysis system.

Given the consideration of the above issues and drawing on the successful application of mass spectrometry to the analysis of XD, in this study, UHPLC-QTOF-MS^E^ was used to detect and analyze XD, ND, ZD, YND, YD, JD, and TD in a unified way and put them into a unified analysis system. On the other hand, the proposal of quality markers (Q-markers) has improved the quality evaluation system for traditional Chinese medicine [11,12]. In terms of Q-markers, the identification of XD and the six other animals’ bile and adulteration analysis will be facilitated if differential Q-markers are found based on the chemometric analysis. Therefore, we carried out the present study to find differential chemical markers for the identification and adulteration analysis of bile classes. 

**Figure 1:**
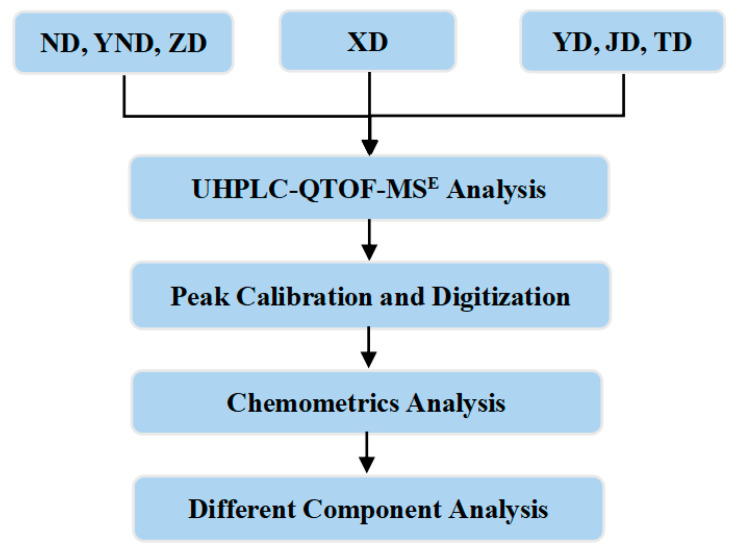
The overall research route of this paper.

In this paper, the overall research route is shown in Figure 1. Firstly, UHPLC-QTOF-MS^E^ technology was utilized to analyze the chemical composition of XD, ND, ZD, YND, YD, JD, and TD under unified analysis conditions. Secondly, the Progenesis QI software (Version 2.3) was used to perform peak position calibration and digitize the mass chromatography of XD, ND, ZD, YND, YD, JD, and TD. Finally, combined with chemometrics, the differences in the chemical components were further discussed, and potential markers that contribute to the differences were explored, in which principal component analysis (PCA), supervised orthogonal partial least squares discriminant analysis (OPLS−DA), and a nonparametric rank sum test were applied to process and analyze the experimental data.

## 2. Results 

### 2.1. UHPLC-QTOF-MS^E^ Analysis

**Figure 2:**
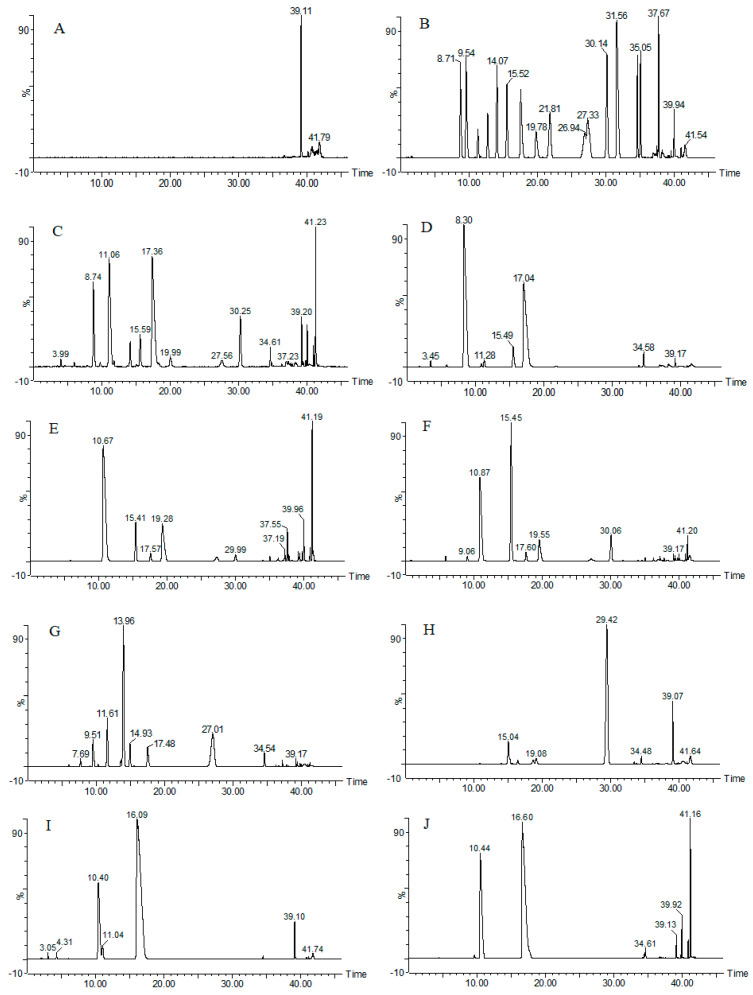
The base-peak chromatogram of blank, mix reference standards, some samples, and the quality control sample ((**A**): blank; (**B**): mixed chemical reference substances; (**C**): QC sample; (**D**): XD; (**E**): YND; (**F**): ND; (**G**): ZD; (**H**): TD; (**I**): YD; (**J**): JD).

Under the sample processing and experimental conditions of 4.3, we obtained the base-peak chromatogram information for XD, ND, ZD, YND, YD, JD, and TD. Chromatograms of the blank, mixed chemical reference substances, some samples, and the quality control sample are shown in Figure 2.

As shown in Figure 2, XD, ND, ZD, YND, YD, JD, and TD showed different chromatograms under unified detection conditions. But the base-peak chromatogram can only present the differences in XD, ND, ZD, YND, YD, JD, and TD as a whole, and it is difficult to establish a characterization relationship between their chemical compositions and the varieties of bile medicines, not to mention that it is impossible to identify the varieties of bile medicines and perform adulteration analysis based on the differences in their chemical compositions. However, multivariate analyses such as PCA and OPLS-DA based on chemometrics can realize a differential analysis of their chemical compositions to find potential chemical markers for the seven kinds of bile medicines. Therefore, in the subsequent section, we quantified the base-peak chromatogram and then performed the multivariate analysis. In addition, as shown in Figure 2B, there were 17 chemical reference substances in the bile acids which were able to meet our identification needs. Confirmed by each single chemical standard substance, the specific information on the bile acid components is detailed in Table 1.

As shown in Table 1, the above chemical components of bile acid help us to clarify the material bases of XD, ND, ZD, YND, YD, JD, and TD. 

Meanwhile, in the UHPLC-QTOF-MS^E^ analysis of XD, ND, ZD, YND, YD, JD, and TD, we explored the sample pretreatment methods, such as solvent extraction with methanol, extraction with water and 50% methanol−water under ultrasound, or heating reflux on the basis of the principle of obtaining more mass spectrometry data information. As a result, methanol extraction showed the optimal number of characteristic peaks and peak shapes, and the information on the compounds obtained was more comprehensive. Moreover, there was essentially no difference between the treatments of ultrasonic extraction and heat reflux. In addition, we examined the extraction situation when the sample powder was extracted by ultrasonication for 15 min, 30 min, and 45 min, respectively, and the results showed that the extraction for 30 min and 45 min was basically the same, which was significantly higher than that for 15 min. Therefore, to optimize the extraction and to save time, we chose ultrasonication for 30 min (power: 500 W, frequency: 40 kHz). In the experimental analysis, we adopted the MS^E^ data-independent acquisition mode to obtain the parent ions and secondary fragment ions of the samples at the same time, thus ensuring more data information and facilitating the subsequent identification analysis [13]. In addition, we investigated the mass spectrum information of XD, ND, ZD, YND, YD, JD, and TD when the collision energies were 10 V~40 V, 10 V~50 V, and 10 V~60 V, respectively. It was distinct that the mass spectrum had the most abundant data information, with the collision energy being 10 V~50 V rather than 10~40 or 10~50 V.

On the other hand, the quality control sample, a mixed sample of XD, ND, ZD, YND, YD, JD, and TD, was taken as the reference to perform peak position correction and data conversion. As we all know, it is difficult to directly place the mass spectrometry data of seven bile medicines into a unified analytical system, which is necessary for chemometric analysis due to their different data volumes and characterizations. However, the quality control sample provided us with the possibility. Using a quality control sample as the reference for peak correction and data transformation, the mass spectrometry data on seven bile medicines can be integrated into a unified analysis system by converting three-dimensional (3D) LC/MS mass spectra into two-dimensional (2D) data matrices and generating peak intensity lists using RT and *m*/*z* data pairs while retaining the data integrity to the greatest extent possible.

### 2.2. Chemometric Analysis 

**Figure 3:**
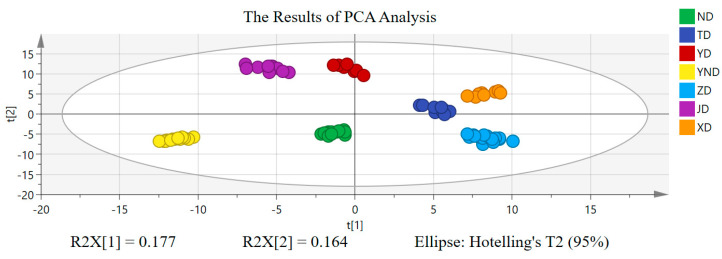
PCA results of XD, ND, ZD, YND, YD, JD, and TD.

All the data information was transformed through QI software (Version 2.3) by taking the quality control sample as a reference [14]. The quantized data were exported into the SIMCA 14.1 software for pattern recognition chemometric analysis. That is to say, the 2D data matrix was analyzed by unsupervised principal component analysis (PCA) and supervised orthogonal partial least squares discriminant analysis (OPLS-DA) to obtain chemical markers of the bile acids for discrimination between XD, ND, ZD, YND, YD, JD, and TD. Principal component analysis was first performed, and score plots of principal component 1 (PC1) versus principal component 2 (PC2) are shown in Figure 3. As a whole, in the PCA of unsupervised learning, XD, ND, ZD, YND, YD, JD, and TD could be effectively and clearly distinguished from each other, which corroborated that their chemical components were indeed different. At the same time, the cumulative interpretation rate of PC1 was 0.177, and that of PC2 was 0.164. 

Principal component analysis (PCA) is a linear dimensionality reduction algorithm and a commonly used data pre-processing method [15]. Its goal is to measure the data variability in terms of variance and to project the higher-dimensional data with higher variability into a lower dimensional space for representation [15,16]. Therefore, in the analysis process, due to the high dimensionality and complexity of mass spectrometry data, we first used principal component analysis for data downscaling. This unsupervised analysis provided us with a differentiation of the seven bile medicines at the overall level, suggesting that XD, ND, ZD, YND, YD, JD, and TD indeed contain different ingredients. 

**Figure 4:**
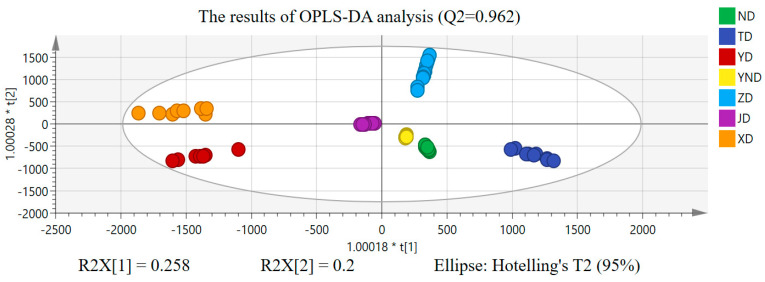
OPLS−DA classification results of XD, ND, ZD, YND, YD, JD, and TD.

Based on the PCA, OPLS−DA was executed to find chemical markers of the bile acids that were responsible for distinguishing XD, ND, ZD, YND, YD, JD, and TD. We divided XD, ND, ZD, YND, YD, JD, and TD data into seven different groups, respectively, to execute OPLS−DA. The results of the OPLS−DA are shown in Figure 4.

**Figure 5:**
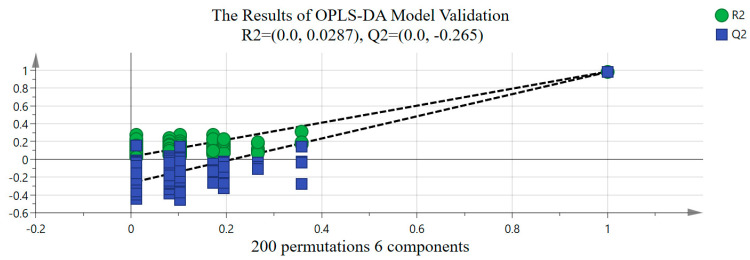
The results of permutation testing in OPLS−DA model.

R2X and R2Y denote the explanatory rate of the OPLS−DA model for the X and Y matrices, respectively. Q2 reflects the prediction ability of the OPLS−DA model. Generally speaking, the closer R2X, R2Y, and Q2 are to 1, the more stable and precise the OPLS−DA model effect is [16,17]. Under OPLS−DA, R2X = 0.953 indicated that the X matrix of independent variables in the OPLS−DA model can reflect 95.3% data changes, and XD, ND, ZD, YND, YD, JD, and TD can be clearly distinguished from each other with small intra-group differences and complete separation of the samples between groups. In addition, R2Y and Q2 of the OPLS−DA model were all higher than 0.960, indicating that the model had a good explaining ability and fitting degree. At the same time, to avoid over-fitting in the OPLS-DA model, we employed permutation testing and cross-validation analysis (CV−ANOVA) using SIMCA 14.1 to assess the model’s reliability. From Figure 5, it can be observed that even after 200 rounds of cross-validation, the response line of model Q2 still intersected with the *x*−axis and had an intercept (−0.265) smaller than 0 with the *y*-axis, indicating that the model was not over-fitted [18]. Additionally, the significance probability value from the cross-validation analysis is *p* = 0 < 0.01, signifying that the OPLS−DA model established in this paper was stable, reliable, and statistically significant.

As we all know, the OPLS−DA method integrates PLS−DA and orthogonal signal correction (OSC) technology, and it can decompose data into predictive and orthogonal components. The predictive component captures category-related information, while the orthogonal component captures category−independent variation. By introducing orthogonal components, OPLS−DA can better account for noise and disturbances in the data and improve the model interpretability, which helps with the exploration of the differential chemical compositions of the seven bile medicines. Therefore, compared to PLS−DA, which is suitable for simple classification problems, OPLS−DA improves the interpretability of the model and is better suited to complex metabolomics data analysis and interpretation. To sum up, we finally chose OPLS−DA.

Based on the above, the variable importance in the projection (VIP) calculated in the OPLS−DA model was used to find variables contributing to distinguishing between XD, ND, ZD, YND, YD, JD, and TD. In principle, the greater the VIP value, the greater the contribution of the component to group classification; moreover, VIP > 1.00 is often considered a commonly used criterion for screening differential components [19,20]. 

**Figure 6:**
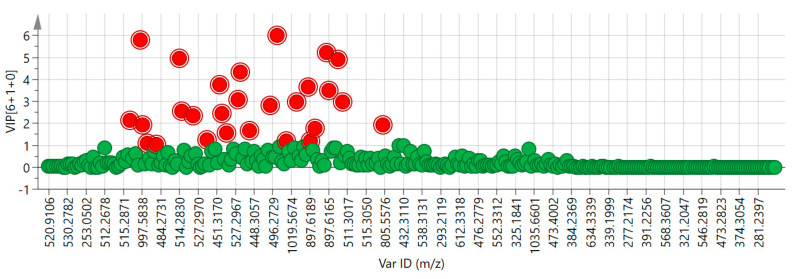
The VIP values of chemical components in OPLS−DA model (Red dots represent chemical constituent ions with VIP > 1.0 and blue dots represent chemical constituent ions with VIP ≤ 1.0)

As shown in Figure 6, there are 312 data points after quantized treatment. Here, each point represents a data point showing the retention time and mass-to-charge ratio (a marker), with the *x*-axis representing the *m*/*z* of the component markers and the *y*-axis representing the VIP value, and there were 27 ions (red spots) whose VIP > 1.00 in the OPLS−DA model. 

As for the 27 potentially different component ions obtained (red spots) whose VIP value was greater than 1.00, the detailed ion information and its VIP values are shown in Table 2.

The above ions in Table 2 represent the important chemical components for distinguishing between XD, ND, ZD, YND, YD, JD, and TD. Furthermore, to verify that the 27 important components obtained based on the chemometrics analysis are indeed significantly different from each other and to make the results of differential components analysis more reliable and accurate on the other hand, the nonparametric rank sum test (the data are not normally distributed) is used as a supplementary test method to verify whether there is a significant difference in the 27 potentially different component ions. The results of the nonparametric tests are detailed in Table S1.

As shown in Table S1, the *p*-values of the 27 ions/neutral ions were all < 0.01, which showed that the 27 potentially different component ions had significant differences among XD, ND, ZD, YND, YD, JD, and TD. The 27 ions that represent these chemical components are expected to be potential marker components for differentiating between XD, ND, ZD, YND, YD, JD, and TD.

Further, based on the results on the chemical reference substances of the bile acids from the UHPLC-QTOF-MS^E^ analysis in Section 2.1, as well as references and database comparisons [21,22,23], we preliminary identified bile acid-like chemical constituents. For example, compound A (8.73 min_498.2887 *m*/*z*, [M−H]^−^) in XD has the highest ionic intensity; at the same time, the ionic strength of compound A in ND, YND, YD, JD, and TD is essentially at baseline levels. In addition, compound A can be detected in ZD, but its ionic strength is far below that in XD. On the other hand, the data pair of (8.73 min_998.5911n, [M+M]; 8.73 min_997.5838 *m*/*z*, [2M−H]^−^) is a dimerization of compound A and can only be detected in XD. Therefore, compound A can be regarded as one of the marker chemical components of XD. After comparison with the chemical reference substances and database comparisons [22], we identified compound A as tauroursodeoxycholic acid (TUDCA). Compound B (14.12 min_448.3053 *m/z*, [M−H]^−^) can be only detected in the ZD samples with a high ionic strength. In addition, the data pair of (14.12 min_898.6250 n [M+M]; 14.12 min_897.6177 *m/z* [2M−H]^−^) is a dimerization of compound B and can also only be detected in ZD. So, compound B can be regarded as distinct within the chemical composition of ZD. After comparison with the chemical reference substances, we identified compound B as Glycohyodeoxycholic acid (GHDCA). The neutral molecules and ions of compound C are (27.28 min_408.2866 n) and (27.28 min_407.2764 *m*/*z*, [M−H]^−^), respectively. This suggested that its molecular formula is C_24_H_40_O_5_, and through comparison with the chemical reference substances and database comparisons, compound C was identified as Cholic acid (CA) and could be detected in YND, JD, and ND with a high ionic strength. At the same time, CA was not detected in the bile of the other animals, with the ionic strength being essentially at baseline levels. Compound D appeared as a subtracted ion [M−H]^−^ at *m*/*z* = 448.3058, and the data pair of (30.25 min_898.6262 n [M+M]; 30.25 min_897.6189 *m*/*z* [2M−H]^−^) is a dimerization of compound D. It could be detected in TD, ND, and YND, rather than in XD, JD, YD, or ZD; moreover, the ionic strength of compound D in TD is more than 10 times the ionic strength of compound D in ND and YND. So, compound D can also be regarded as one of the marker chemical components of TD. Further, after comparison with the chemical reference substances and database comparisons [22], we identified compound D as Glycohyodeoxycholic acid (GDCA). In addition, compound E (15.48 min_464.3006 *m*/*z*, [M−H]^−^) and its dimer (15.48 min_930.6146 n [M+M]; 15.48 min_929.6074 *m*/*z*, [2M−H]^−^) were identified as Glycocholic acid (GCA) through comparing with the chemical reference substances. Similar to the GDCA situation, GCA could only be detected in TD, ND, and YND, but the ionic strength of GCA in ND is more than 2 times the ionic strength of GCA in TD and YND. So, GCA can be regarded as one of the marker chemical components of ND. 

As mentioned above, we preliminary identified 10 bile acid-like chemical constituents whose VIP > 1.0 by comparing them with the chemical reference substances and references and database comparisons. These compounds may be potential difference marker components to distinguish between XD, ND, ZD, YND, YD, JD, and TD. The detailed information on the 10 bile acid-like chemical constituents is shown in Table 3.

In addition, as shown in Table S1, there were some unknown chemical components that could distinguish between XD, ND, ZD, YND, YD, JD, and TD. For example, chemical component F (13.75 min_447.2972 n, 13.75 min_446.2899 *m*/*z*, [M−H]^−^) could only be detected in the ZD samples, while chemical component G (18.82 min_555.3085 *m*/*z*) could only be detected in the TD samples. In addition, chemical component H (9.11 min_462.2856 *m*/*z*) had the highest ionic strength in ND. At the same time, the ionic strength of compound H in XD, YND, YD, JD, ZD, and TD is essentially at baseline levels. That is to say, chemical components F, G, and H can be regarded as potential marker chemical components for ZD, TD, and ND.

To sum up, based on the chemometric analysis and nonparametric test statistics, we further summarized the potential chemical composition markers that may be used to distinguish between XD, ND, ZD, YND, YD, JD, and TD. The potential chemical composition markers for distinguishing ND, XD, YND, YD, JD, ZD, and TD are shown in Table 4. 

In the differential component analysis, the VIP values and the nonparametric rank sum test were used to screen the differential chemical components. The VIP value represents the importance of the component variables in the OPLS−DA model, and the higher its value, the more important a component is for group differentiation. Similarly, VIP > 1.0 was set as the threshold for component marker screening in this study. Due to the non-normal distribution of the data, the variance analysis was performed using a nonparametric rank sum test to verify whether there were significant group differences in the chemical components. The combination of VIP values and nonparametric rank-sum tests ensured the accuracy and reliability of the analysis results.

## 3. Discussion

In this experiment, a method based on UHPLC-Q-TOF-MS^E^ combined with chemometrics was established to analyze the differences in the chemical components of ND, XD, YND, YD, JD, ZD, and TD, to identify the chemical components of bile acids with significant differences, and to provide a reliable and accurate method for the analysis of XD and the identification of the differences in the chemical components of the bile acids. The characteristic peaks were extracted by mass spectrometry analysis, peak matching, peak alignment, and filtering noise treatment, and the metabolic profiles and chemical compositional variability were analyzed in general by using PCA and OPLS-DA for data processing. This process relies on the representativeness of the samples and the accuracy of the data. So, in the analysis process, by taking TUDCA, TCDCA, TCA, and GCA as the targets, we used the XD and ND samples to investigate the precision and accuracy. The results are shown in Tables S2 and S3, and they show that the instrument has good precision and accuracy, with RSD < 0.005% and RE < 0.005%. At the same time, by taking TUDCA, TCDCA, GHDCA, and GCA as the targets, we used the XD, ZD, and ND samples (six samples of each bile medicines) to investigate the repeatability. The results are shown in Table S4, and they show that the method has good reproducibility, with RSD < 0.002%. In addition, we used LE to correct the mass axis in real time to ensure the accuracy of the sample data. On the other hand, the samples come from the National Institutes for Food and Drug Control, which guarantees the authenticity of bile medicines. Their collection must be strictly standardized in terms of origin and harvesting, and the species and origin are completely accurate and verified by experts, in line with the quality requirements. Therefore, the samples with different origins are representative, and further based on the representative samples from different sources, we can control the chemical composition differences to the greatest extent and realize accurate chemometrics analysis. However, it is undeniable that individual or origin differences do exist in Chinese medicine samples. So, follow-up studies may require us to collect a larger sample size. Moreover, for compounds F, G, and H, we do not know their chemical structure, and this needs further exploration. 

On the other hand, we used the MS^E^ mode of data acquisition, i.e., the simultaneous acquisition of primary and secondary ion information. However, in the data acquisition, fewer fragments of secondary ions were collected, and the chemical compositions of the bile acids mainly existed in the form of [M−H]^−^ and [M+HCOO]^−^, which are consistent with the mass spectrometry results stored in the HMDB database [22,23]. Fortunately, we have the chemical reference substances for bile acids to help us to identity bile acid components. 

For the obtained potential chemical composition markers, tauroursodeoxycholic acid (8.73 min_498.2887 *m*/*z*) is distinct within the chemical composition of XD. Glycohyodeoxycholic acid (14.12 min_448.3053 *m*/*z*) and chemical component F (13.75 min_446.2899 *m/z*) are distinct within the chemical composition of ZD. Glycodeoxycholic acid (30.22 min_448.3058 *m*/*z*) and chemical component G (18.82 min_555.3085 *m*/*z*) are recognizable as part of the chemical composition of TD. In addition, chemical component H (9.11 min_462.2856 *m*/*z*) is distinct within the chemical composition of ND. These chemical constituents help to enhance identification analysis and quality control for bile medicines. At the same time, for components that can be detected in a wide range of bile medicines, they can be used as complementary tests. For example, Cholic acid can be detected in YND, JD, and ND, and Taurodeoxycholic acid can be detected in ND, TD, and YND but not detected in XD. So, we could specify that Cholic acid should not be detected in XD, and so on. In addition, for non-proprietary bile acid-like chemical components, perhaps their permutations and combinations or exclusionary method can improve the matching credibility. For example, for bile medicines, if Cholic acid and Glycocholic acid can be detected in an unknown sample at the same time, we can identify this sample as ND or adulterated with ND. 

In this paper, based on UHPLC-QTOF-MS^E^ and multivariate statistical analysis, we carried out a comparative analysis of ND, XD, YND, YD, JD, ZD, and TD. For the chemical components with VIP > 1.0, 10 compounds were identified and may be potential quality control markers to distinguish between these bile medicines. It is beneficial to strengthen the quality control for bile medicines and crack down on fake and shoddy products, thus standardizing the market and promoting the healthy and sustainable development of the Chinese medicine industry. However, on the other hand, from the point of view of compositional identification, this means that we do not know what most compounds are. It is well known that the composition of traditional Chinese medicine is very complex, containing thousands of compounds, and the identification of compounds is time-consuming, laborious, and not always accurate. Therefore, rational utilization of information on unknown components in traditional Chinese medicines to facilitate quality control of traditional Chinese medicines may be a direction for future research and development.

## 4. Materials and Methods

### 4.1. Herbal Materials and Chemical Reference Substances 

A total of 80 batches of materials, including XD (8 batches of materials), JD (11 batches of materials), ZD (17 batches of materials), YND (15 batches of materials), YD (7 batches of materials), TD (9 batches of materials), and ND (13 batches of materials), were collected from the National Institutes for Food and Drug Control. All the samples were identified by the laboratory and met the requirements. All the samples were stored in a cool and dry place. The chemical reference substances were purchased from National Institutes for Food and Drug Control and Shanghai Yuanye Bio-Technology Co., Ltd. 

### 4.2. Reagent Materials

The methanol (MS grade, Lot: ED341-CN) was purchased from Honeywell Trading Co., Ltd. of Shanghai, China. The acetonitrile (MS grade, Lot: 222372) was purchased from Thermo Fisher Scientific Technology Co., Ltd. of Shanghai, China. Mass spectrometry-grade formic acid (Lot: L1670) was purchased from Honeywell Trading Co., Ltd. of Shanghai, China. Ultrapure water (Lot: GB 19298) was purchased from Watsons Food and Beverage Co., Ltd. of Guangzhou, China.

### 4.3. Sample Pretreatment and UHPLC-QTOF-MS^E^ Analysis

A total of 10.00 mg of each chemical reference substance was weighed precisely, and the solution was fixed to 200 mL; then, 1.00 mL of each chemical reference substance was pipetted and diluted to 100 mL again to make a mixed standard solution with a concentration of 500 ng/mL for identification of its chemical composition. 

The specific procedure for sample pretreatment of the bile medicines is as follows: First, accurately weigh 25.00 mg of dried powder of the bile medicine, and take the dried powder of the bile medicine and place it in a 50 mL tapered bottle with a plug, respectively; then, accurately add 25.00 mL of mass spectroscopy methanol with a pipette into the tapered bottle to perform the ultrasound for 0.5 h (power: 500 W, frequency: 40 kHz); finally, take it out and cool it at room temperature, as well as filtering it with a 0.22 μm organic filter membrane to obtain the samples to be analyzed. The samples were stored at 4 °C in a refrigerator before the UHPLC-QTOF-MS^E^ analysis. In addition, the quality control sample was a mixed sample of ND, XD, YND, YD, JD, ZD, and TD. 

The UHPLC-QTOF-MS^E^ analysis were performed using liquid chromatography tandem time-of-flight mass spectrometry on a Waters Xevo G2-XS QTof (Waters in Milford, Massachusetts, USA) [24,25]. Chromatographic separations were conducted on a Waters Acquity UPLC BEH-C_18_ (2.1 mm × 100 mm, 1.7 μm) chromatographic column (Lot: 186002352 Waters, USA). For the analysis of ND, XD, YND, YD, JD, ZD, and TD, the column temperature was programmed at 35 °C. The mobile phases were methanol (A)–acetonitrile (B)–5% ammonium formate solution, and the gradient elution conditions are shown in Table 5. 

The injection volume was 2.0 μL. On the other hand, in this study, an ESI-positive ionization mode was used for detection and analysis, and the MS^E^ data acquisition method was used, in which the data acquisition rate was set to 0.2 s; the scanning range of *m/z* was 100~1500; the collision gas was high-purity argon; and the real-time mass axis calibration solution (lock mass) was Leucine Enkephalin (LE), whose concentration was 300 ng/mL. The following additional settings were implemented: capillary: 3.0 kV; sampling cone: 40 V; source offset: 80 V; desolvation temperatures: 450 °C; desolvation gas: 900 L/h, collision energy: 10 V~50 V; source temperatures: 120 °C. Before the sample analysis, calibration of the mass axis and lock mass was performed.

### 4.4. Data Processing and Analysis

The mass spectrometry information on ND, XD, YND, YD, JD, ZD, and TD was processed by Progenesis QI software (Version 2.3) with the following parameters: type of machine: high-resolution mass spectrometer; ionization polarity: positive; retention time: 1.00–40.00 min; peak picking limits: automatic; RT window: 0.2 min. We obtained the quantized data, including the retention time, mass-to-charge ratio, and ionic strength, and converted the 3D LC/MS data into a 2D data matrix, expressed by taking the retention time/mass–charge ratio as the abscissa and ionic strength as the ordinate, respectively. For the quantized data analysis, Simca P 14.1 (Umetrics, Umea, Sweden) was used for the data processing and data analysis, in which an unsupervised pattern recognition method–principal component analysis (PCA) and a supervised learning method (PLS−DA) were adopted [26,27,28]. Firstly, the PCA method was employed to visualize the global variance in the datasets and find outliers. Then the PLS−DA method was applied to maximize the covariance between the X variable measured data and the Y variable predictive classifications simultaneously to remove non-correlated variation between the X variables and the Y variables. The Hotelling’s T2 region, an ellipse in the score plot, provided the 95% confidence interval of the modeled variation. R2 and Q2 values were used to evaluate the model quality. R2 indicates goodness of fit and refers to the proportion of variance in the data explained by the models, with the Q2 being defined as the proportion of variance in the data predictable by the model and indicating the predictability. In addition, multivariate variable importance in projection (VIP) and differential statistical analysis were used to search for and verify the differential chemical components [29].

## 5. Conclusions

In this paper, a UHPLC/QTOF-MS^E^ method and chemometrics were successfully applied to differentiate between ND, XD, YND, YD, JD, ZD, and TD. The method incorporated them into a unified analytical system and achieved efficient and rapid differential compositional marker analysis at a low concentration level. The method was efficient and rapid in accurately and reliably distinguishing between ND, XD, YND, YD, JD, ZD, and TD and provided a scientific basis for avoiding confusion, adulterants, and the misuse of bile medicines. In identifying the constituents, through database retrieval and chemical reference substance comparison, combined with multivariate statistical analysis, 10 chemical constituents were identified, such as tauroursodeoxycholic acid, Glycohyodeoxycholic acid, Glycodeoxycholic acid, and chemical component H (9.11_462.2856 *m/z*), which may be used as chemical markers for XD, ZD, TD, and ND. Based on the potential chemical composition markers obtained in this paper, it is beneficial to strengthen quality control for bile medicines.

## Figures and Tables

**Table 1:** The specific information on 17 bile acid components.

Compositions	Ions	Abbreviation	Compositions	Ions	Abbreviation
Tauroursodeoxycholic acid	8.71 min_*m*/*z* 498.29	TUDCA	Taurocholic acid	11.08 min_*m*/*z* 514.28	TCA
Taurohyodeoxycholic acid	9.54 min_*m*/*z* 498.30	THDCA	Glycocholic acid	15.52 min_*m*/*z* 464.30	GCA
Glycoursodeoxycholic acid	12.73 min_*m*/*z* 498.30	GUDCA	Taurodeoxycholic acid	19.78 min_*m*/*z* 498.29	TDCA
Glycohyodeoxycholic acid	14.07 min_*m*/*z* 448.30	GHDCA	Ursodeoxycholic acid	21.81 min_*m*/*z* 437.30	UDCA
Taurochenodeoxycholic acid	16.90 min_*m*/*z* 498.29	TCDCA	Hyodeoxycholic acid	26.72 min_*m*/*z* 437.29	HDCA
Glycochenodeoxycholic acid	26.94 min_*m*/*z* 448.30	GCDCA	Cholic acid	27.33 min_*m*/*z* 407.30	CA
Glycodeoxycholic acid	30.14 min_*m*/*z* 448.30	GDCA	Taurolithocholic acid	31.56 min_*m*/*z* 482.29	TLCA
Chenodeoxycholic acid	34.57 min_*m*/*z* 448.30	CDCA	Deoxycholic acid	35.05 min_*m*/*z* 391.28	DCA
Lithocholic acid	37.67 min_*m*/*z* 448.30	LCA	—	—	—

**Table 2:** The VIP values of 27 potentially different component ions.

Ions/Neutral Mass	VIP	Ions/Neutral Mass	VIP
16.94_498.2881 *m*/*z*	6.00	14.12_897.6177 *m*/*z*	2.45
8.73_498.2887 *m*/*z*	5.82	11.75_464.3007 *m*/*z*	2.34
29.63_448.3053 *m*/*z*	5.23	8.46_997.5836 *m*/*z*	2.13
11.02_514.2830 *m*/*z*	4.98	8.73_997.5838 *m*/*z*	1.92
30.22_448.3058 *m*/*z*	4.92	34.60_437.2897 *m*/*z*	1.91
15.48_464.3006 *m*/*z*	4.33	27.28_407.2764 *m*/*z*	1.79
14.12_448.3053 *m*/*z*	3.76	15.73_496.2733 *m*/*z*	1.66
27.17_448.3059 *m*/*z*	3.64	15.06_446.2897 *m*/*z*	1.57
29.64_897.6165 *m*/*z*	3.52	13.75_446.2899 *m*/*z*	1.25
15.48_929.6074 *m*/*z*	3.07	18.82_555.3085 *m*/*z*	1.22
19.55_498.2885 *m*/*z*	2.96	27.20_897.6189 *m*/*z*	1.19
30.25_897.6189 *m*/*z*	2.96	9.11_462.2856 *m*/*z*	1.10
16.82_997.5822 *m*/*z*	2.79	9.79_448.3058 *m*/*z*	1.04
11.02_1029.5717 *m*/*z*	2.55	—	—

**Table 3:** The detailed information on 10 bile acid-like chemical constituents.

Compositions	Molecular Formula	Ions/Neutral Mass	Ionic Forms	VIP
Taurochenodeoxycholic acid	C26H45NO6S	16.94_498.2881 *m*/*z*	[M−H]^−^	6.00
Tauroursodeoxycholic acid	C26H45NO6S	8.73_498.2887 *m*/*z*	[M−H]^−^	5.82
Taurocholic acid	C26H45NO7S	11.02_514.2830 *m*/*z*	[M−H]^−^	4.98
Glycodeoxycholic acid	C26H43NO5	30.22_448.3058 *m*/*z*	[M−H]^−^	4.92
Glycocholic acid	C26H43NO6	15.48_464.3006 *m*/*z*	[M−H]^−^	4.33
Glycohyodeoxycholic acid	C26H43NO5	14.12_448.3053 *m*/*z*	[M−H]^−^	3.76
Glycochenodeoxycholic acid	C26H43NO5	27.17_448.3059 *m*/*z*	[M−H]^−^	3.64
Taurodeoxycholic acid	C26H44NO6S	19.55_498.2885 *m*/*z*	[M−H]^−^	2.96
Chenodeoxycholic acid	C24H40O4	34.60_437.2897 *m*/*z*	[M+HCOO]^−^	1.91
Cholic acid	C24H40O5	27.28_407.2764 *m*/*z*	[M−H]^−^	1.79

**Table 4:** The potential chemical composition markers.

Compositions	Molecular Formula	Ions/Neutral Mass	Ionic Forms	Specific Attribution
Tauroursodeoxycholic acid	C26H45NO6S	8.73_498.2887 *m*/*z*	[M−H]^−^	XD
Glycohyodeoxycholic acid	C26H43NO5	14.12_448.3053 *m*/*z*	[M−H]^−^	ZD
Cholic acid	C24H40O5	27.28_407.2764 *m*/*z*	[M−H]^−^	YND, JD, ND
Glycodeoxycholic acid	C26H43NO5	30.22_448.3058 *m*/*z*	[M−H]^−^	TD
Glycocholic acid	C26H43NO6	15.48_464.3006 *m*/*z*	[M−H]^−^	ND, TD
Glycochenodeoxycholic acid	C26H43NO5	27.17_448.3059 *m*/*z*	[M−H]^−^	ND, ZD
Taurodeoxycholic acid	C26H44NO6S	19.55_498.2885 *m*/*z*	[M−H]^−^	ND, TD, YND
Chemical component F	—	13.75_446.2899 *m*/*z*	[M−H]^−^	ZD
Chemical component G	—	18.82_555.3085 *m*/*z*	[M−H]^−^	TD
Chemical component H	—	9.11_462.2856 *m*/*z*	[M−H]^−^	ND

**Table 5:** The gradient elution program.

Time	Flow (mL/min)	%A	%B	%C
0	0.3	14.0	23.0	63.0
15	0.3	24.0	29.0	47.0
20	0.3	20.0	29.0	51.0
25	0.3	15.0	29.0	56.0
30	0.3	30.0	35.0	35.0
40	0.3	25.0	72.0	3.0
41	0.3	14.0	23.0	63.0
45	0.3	14.0	23.0	63.0

## Data Availability

All the data information can be obtained from the paper or by contacting the corresponding author.

## Supplementary Materials

The following supporting information can be downloaded at https://www.mdpi.com/article/10.3390/molecules29133144/s1. Table S1: the results of non-parametric statistical tests; Table S2: the results of precision investigation; Table S3: the results of accuracy investigation; Table S4: the results of repeatability investigation.
