# Supplementary material for: A Comprehensive Analysis of Fel Ursi and Its Common Adulterants Based on UHPLC-QTOF-MSE and Chemometrics
Source: Molecules. 2024 Jul 2;29(13):3144. doi: 10.3390/molecules29133144 (PMC11243315; doi:10.3390/molecules29133144)
Supplement: Supplementary file 1 [file molecules-29-03144-s001.zip › molecules-3063658-supplementary.pdf]

Table S1 the results of non-parametric statistical tests

Ions	Condition median M(P25，P75)							*H*-value	*p*
	JD(*n*=11)	ND(*n*=13)	TD(*n*=9)	XD(*n*=8)	YD(*n*=7)	YND(*n*=15)	ZD(*n*=17)		
16.94_498.2881 m/z	209778.000(188487.0,232592.0)	28394.200(26274.5,29892.3)	7.067(0.0,21.9)	584938.000(502364.0,617388.8)	934588.000(913596.0,1040000.0)	12417.300(12188.2,12920.3)	69637.900(62487.8,79844.0)	75.876	0.000**
8.73_498.2887m/z	194.780(165.3,218.0)	88.455(63.0,106.1)	15.291(2.9,171.7)	809433.500(738115.5,873502.5)	89.142(77.6,158.3)	33.091(27.1,38.0)	65965.300(59720.7,76191.4)	67.678	0.000**
29.63_448.3053 m/z	0.000(0.0,0.0)	2042.610(1465.1,4708.8)	14960.400(1124.5,1380000.0)	31.323(0.0,81.4)	0.000(0.0,0.0)	1123.380(1001.8,1524.3)	0.000(0.0,0.0)	68.089	0.000**
11.02_514.2830 m/z	47484.900(42741.6,52769.1)	272502.000(252963.0,287529.5)	2105.680(1117.4,3731.1)	16498.050(13198.8,25047.8)	497379.000(480747.0,546535.0)	227301.000(215131.0,233680.0)	27.444(23.4,34.1)	76.064	0.000**
30.22_448.3058 m/z	0.000(0.0,0.0)	71059.800(64152.8,75809.9)	738256.000(107276.7,834048.5)	0.000(0.0,0.0)	0.000(0.0,0.0)	8850.780(8376.7,9499.7)	287.075(244.1,322.5)	76.364	0.000**
15.48_464.3006 m/z	0.000(0.0,0.0)	220994.000(207551.0,240534.0)	110098.000(93643.7,204198.0)	0.000(0.0,0.0)	0.000(0.0,0.0)	38780.600(37150.1,40042.3)	331.547(297.9,391.7)	71.869	0.000**
14.12_448.3053 m/z	0.000(0.0,0.0)	0.000(0.0,0.0)	0.000(0.0,34.4)	0.000(0.0,0.0)	0.000(0.0,0.0)	0.000(0.0,0.0)	232939.000(214427.5,271562.5)	72.507	0.000**
27.17_448.3059 m/z	0.000(0.0,0.0)	15154.400(14521.4,15616.3)	345.989(58.2,978.0)	0.000(0.0,0.0)	0.000(0.0,0.0)	368.292(343.7,381.7)	219631.000(201036.0,255292.0)	74.8	0.000**
29.64_897.6165 m/z	0.000(0.0,0.0)	2.805(0.0,99.7)	12.739(0.0,633795.5)	0.000(0.0,0.0)	0.000(0.0,0.0)	0.000(0.0,0.0)	0.000(0.0,0.0)	36.67	0.000**
15.48_929.6074 m/z	0.000(0.0,0.0)	89183.900(82839.2,91869.6)	7136.460(3784.7,9959.0)	0.000(0.0,0.0)	0.000(0.0,0.0)	4487.090(4387.8,4635.4)	0.000(0.0,0.0)	74.187	0.000**

19.55_498.2885 m/z	2.211(1.8,6.5)	94782.900(88998.8,98825.2)	47780.600(28635.2,88008.1)	1544.495(1268.8,1755.2)	289.491(243.2,383.3)	85163.700(82030.4,88035.7)	86.754(41.3,242.7)	70.381	0.000**
30.25_897.6189 m/z	0.000(0.0,0.0)	4561.970(4091.7,5049.5)	239279.000(4258.2,351203.5)	0.000(0.0,0.0)	0.000(0.0,0.0)	6.207(3.1,9.5)	0.000(0.0,0.0)	65.899	0.000**
16.82_997.5822 m/z	34007.600(31159.3,37135.9)	32.662(4.0,64.3)	0.000(0.0,0.0)	98063.550(83022.0,113809.3)	203597.000(196133.0,218021.0)	8.951(6.1,11.7)	1691.690(1370.4,1786.6)	74.175	0.000**
11.02_1029.5717 m/z	1498.960(1330.3,1601.2)	39891.600(38915.8,41176.3)	0.000(0.0,0.0)	0.000(0.0,0.0)	111292.000(103043.0,115969.0)	48218.100(46205.7,50156.6)	0.000(0.0,0.0)	77.797	0.000**
14.12_897.6177 m/z	0.000(0.0,0.0)	0.000(0.0,0.0)	0.000(0.0,0.0)	0.000(0.0,0.0)	0.000(0.0,0.0)	0.000(0.0,0.0)	99825.000(89713.2,114432.5)	77.523	0.000**
11.75_464.3007 m/z	0.000(0.0,0.0)	0.000(0.0,0.0)	0.000(0.0,0.0)	0.000(0.0,0.0)	0.000(0.0,0.0)	0.000(0.0,0.0)	90080.900(82556.1,105411.5)	77.523	0.000**
8.46_997.5836m/z	0.000(0.0,0.0)	0.000(0.0,0.0)	0.000(0.0,0.0)	151035.500(112305.3,166753.5)	0.000(0.0,0.0)	0.000(0.0,0.0)	0.000(0.0,0.0)	78.713	0.000**
8.73_997.5838 m/z	0.000(0.0,0.0)	0.000(0.0,0.0)	0.000(0.0,0.0)	63252.200(41385.4,102058.8)	0.000(0.0,0.0)	0.000(0.0,0.0)	0.000(0.0,70.8)	58.237	0.000**
34.60_437.2897 m/z	28551.800(26122.3,31170.3)	1149.370(1113.5,1173.2)	0.000(0.0,0.0)	63758.245(206.7,158173.5)	12380.500(11463.3,14025.8)	301.510(286.9,315.4)	26083.500(24403.8,32010.8)	59.69	0.000**
27.28_407.2764 m/z	3525.170(3148.2,3819.0)	13005.500(12512.0,13235.9)	0.000(0.0,0.0)	592.609(246.0,791.9)	0.000(0.0,0.0)	25456.300(24910.7,26427.5)	0.000(0.0,0.0)	77.282	0.000**
15.73_496.2733 m/z	143.890(123.3,606.5)	0.000(0.0,0.0)	0.000(0.0,0.0)	61604.050(30893.4,101832.6)	2646.400(2310.6,2784.5)	0.000(0.0,0.0)	0.000(0.0,0.0)	76.739	0.000**
15.06_446.2897 m/z	0.000(0.0,0.0)	0.000(0.0,2.1)	0.000(0.0,12577.6)	0.000(0.0,0.0)	0.000(0.0,0.0)	0.000(0.0,0.0)	51167.100(19598.3,59631.1)	63.926	0.000**

13.75_446.2899 m/z	0.000(0.0,0.0)	0.000(0.0,0.0)	0.000(0.0,0.0)	0.000(0.0,0.0)	0.000(0.0,0.0)	0.000(0.0,0.0)	21557.000(18864.3,45061.3)	77.523	0.000**
18.82_555.3085 m/z	0.000(0.0,0.0)	0.000(0.0,0.0)	1930.190(213.4,84431.1)	0.000(0.0,0.0)	0.000(0.0,0.0)	0.000(0.0,0.0)	0.000(0.0,0.0)	68.995	0.000**
27.20_897.6189 m/z	0.000(0.0,0.0)	0.000(0.0,0.0)	0.000(0.0,0.0)	0.000(0.0,0.0)	0.000(0.0,0.0)	0.000(0.0,0.0)	23960.400(20796.8,26889.4)	77.523	0.000**
9.11_462.2856m/z	0.000(0.0,0.0)	10188.900(9828.3,10513.9)	446.098(0.0,1598.8)	0.000(0.0,0.0)	0.000(0.0,0.0)	17.515(14.8,23.3)	1939.090(1771.7,2347.5)	71.636	0.000**
9.79_448.3058m/z	0.000(0.0,0.0)	0.000(0.0,0.0)	0.000(0.0,0.0)	0.000(0.0,0.0)	0.000(0.0,0.0)	0.000(0.0,0.0)	18102.400(16209.5,20495.7)	77.523	0.000**

* p<0.05 ** p<0.01

Table S2 the results of precision investigation

Numbers	TUDCA	TCDCA	TCA	GCA
1	498.29	498.30	514.28	464.30
2	498.30	498.30	514.29	464.30
3	498.29	498.30	514.30	464.31
4	498.30	498.30	514.29	464.31
5	498.29	498.29	514.28	464.30
6	498.29	498.29	514.28	464.30
Average	498.293	498.297	514.287	464.303
S	0.005	0.005	0.008	0.005
RSD (%)	0.001	0.001	0.002	0.001

Table S3 the results of accuracy investigation

Numbers	TUDCA	TCDCA	TCA	GCA
1	498.29	498.30	514.28	464.30
2	498.30	498.30	514.29	464.30
3	498.29	498.30	514.30	464.31
4	498.30	498.30	514.29	464.31
5	498.29	498.29	514.28	464.30
6	498.29	498.29	514.28	464.30
Average	498.293	498.297	514.287	464.303
Theoretical value	498.300	498.300	514.300	464.300
RE (%)	0.001	0.001	0.003	0.001

Table S4 the results of repeatability investigation

Samples	TUDCA	TCDCA	GHDCA	GCA
1	498.29	498.29	514.28	464.30
2	498.29	498.29	514.28	464.30
3	498.29	498.30	514.28	464.31
4	498.30	498.30	514.29	464.31
5	498.28	498.29	514.28	464.30
6	498.29	498.29	514.27	464.30
Average	498.290	498.293	514.280	464.303
S	0.006	0.005	0.006	0.005
RSD (%)	0.001	0.001	0.001	0.001
